# Supplementary material for: HIVprotI: an integrated web based platform for prediction and design of HIV proteins inhibitors
Source: J Cheminform. 2018 Mar 9;10:12. doi: 10.1186/s13321-018-0266-y (PMC5845081; doi:10.1186/s13321-018-0266-y)
Supplement: Supplementary file 2 — Additional file 2. Source code of HIVProtI web server. [file 13321_2018_266_MOESM2_ESM.zip › HIVprotI_Source-code/hivprotI/weka-3-6-6/doc/weka/classifiers/trees/j48/BinC45ModelSelection.html]

BinC45ModelSelection


---


|  |  |  |
| --- | --- | --- |
| **Overview** \| **Package** \| **Class** \| **Tree** \| **Deprecated** \| **Index** \| **Help** \| **Weka's home** | |  |
| PREV CLASS   **NEXT CLASS** | **FRAMES**    **NO FRAMES**     **All Classes** |  |
| SUMMARY: NESTED \| FIELD \| CONSTR \| METHOD | DETAIL: FIELD \| CONSTR \| METHOD |  |


---


## weka.classifiers.trees.j48 Class BinC45ModelSelection

```
java.lang.Object
  weka.classifiers.trees.j48.ModelSelection
      weka.classifiers.trees.j48.BinC45ModelSelection
```

**All Implemented Interfaces:**: java.io.Serializable, RevisionHandler

---

``` public class BinC45ModelSelection extends ModelSelection ```

Class for selecting a C4.5-like binary (!) split for a given dataset.

**Version:**
:   $Revision: 1.11 $

**Author:**
:   Eibe Frank (eibe@cs.waikato.ac.nz)

**See Also:**: Serialized Form

---

**Constructor Summary**

`BinC45ModelSelection(int minNoObj,
Instances allData)`
  
          Initializes the split selection method with the given parameters.


| **Method Summary** | |
| --- | --- |
| `void` | `cleanup()`             Sets reference to training data to null. |
| `java.lang.String` | `getRevision()`             Returns the revision string. |
| `ClassifierSplitModel` | `selectModel(Instances data)`             Selects C4.5-type split for the given dataset. |
| `ClassifierSplitModel` | `selectModel(Instances train, Instances test)`             Selects C4.5-type split for the given dataset. |

**Methods inherited from class java.lang.Object**

`equals, getClass, hashCode, notify, notifyAll, toString, wait, wait, wait`

**Constructor Detail**

### BinC45ModelSelection

```
public BinC45ModelSelection(int minNoObj,
                            Instances allData)
```

:   Initializes the split selection method with the given parameters.

    **Parameters:**: `minNoObj` - minimum number of instances that have to occur in at least two subsets induced by split: `allData` - FULL training dataset (necessary for selection of split points).


**Method Detail**

### cleanup

```
public void cleanup()
```

:   Sets reference to training data to null.

---


### selectModel

```
public final ClassifierSplitModel selectModel(Instances data)
```

:   Selects C4.5-type split for the given dataset.

    :   **Specified by:**: `selectModel` in class `ModelSelection`

---


### selectModel

```
public final ClassifierSplitModel selectModel(Instances train,
                                              Instances test)
```

:   Selects C4.5-type split for the given dataset.

    :   **Overrides:**: `selectModel` in class `ModelSelection`

---


### getRevision

```
public java.lang.String getRevision()
```

:   Returns the revision string.

    :   **Returns:**: the revision


---


|  |  |  |
| --- | --- | --- |
| **Overview** \| **Package** \| **Class** \| **Tree** \| **Deprecated** \| **Index** \| **Help** \| **Weka's home** | |  |
| PREV CLASS   **NEXT CLASS** | **FRAMES**    **NO FRAMES**     **All Classes** |  |
| SUMMARY: NESTED \| FIELD \| CONSTR \| METHOD | DETAIL: FIELD \| CONSTR \| METHOD |  |


---
